# Mathematical modeling of endovenous laser treatment (ELT)

**DOI:** 10.1186/1475-925X-5-26

**Published:** 2006-04-25

**Authors:** Serge R Mordon, Benjamin Wassmer, Jaouad Zemmouri

**Affiliations:** 1 INSERM (French National Institute of Health and Medical Research) IFR 114, Lille University Hospital, Lille, France; 2 Osyris SA, Hellemmes, France

## Abstract

**Background and objectives:**

Endovenous laser treatment (ELT) has been recently proposed as an alternative in the treatment of reflux of the Great Saphenous Vein (GSV) and Small Saphenous Vein (SSV). Successful ELT depends on the selection of optimal parameters required to achieve an optimal vein damage while avoiding side effects. Mathematical modeling of ELT could provide a better understanding of the ELT process and could determine the optimal dosage as a function of vein diameter.

**Study design/materials and methods:**

The model is based on calculations describing the light distribution using the diffusion approximation of the transport theory, the temperature rise using the bioheat equation and the laser-induced injury using the Arrhenius damage model. The geometry to simulate ELT was based on a 2D model consisting of a cylindrically symmetric blood vessel including a vessel wall and surrounded by an infinite homogenous tissue. The mathematical model was implemented using the Macsyma-Pdease2D software (Macsyma Inc., Arlington, MA, USA). Damage to the vein wall for CW and single shot energy was calculated for 3 and 5 mm vein diameters. In pulsed mode, the pullback distance (3, 5 and 7 mm) was considered. For CW mode simulation, the pullback speed (1, 2, 3 mm/s) was the variable. The total dose was expressed as joules per centimeter in order to perform comparison to results already reported in clinical studies.

**Results:**

In pulsed mode, for a 3 mm vein diameter, irrespective of the pullback distance (2, 5 or 7 mm), a minimum fluence of 15 J/cm is required to obtain a permanent damage of the intima. For a 5 mm vein diameter, 50 J/cm (15W-2s) is required. In continuous mode, for a 3 mm and 5 mm vein diameter, respectively 65 J/cm and 100 J/cm are required to obtain a permanent damage of the vessel wall. Finally, the use of different wavelengths (810 nm or 980 nm) played only a minor influence on these results.

**Discussion and conclusion:**

The parameters determined by mathematical modeling are in agreement with those used in clinical practice. They confirm that thermal damage of the inner vein wall (tunica intima) is required to achieve the tissue alterations necessary in order to lead the vein to permanent occlusion. However, in order to obtain a high rate of success without adverse events, the knowledge of the vein diameter after tumescent anesthesia is recommended in order to use the optimal energy. As clearly demonstrated by our calculations, both pulsed and continuous mode operations of the laser can be efficient. An interesting observation in our model is that less amount of energy is required in pulsed mode than in continuous mode. Damaging the vein sequentially along its entire length may lead to permanent occlusion. However, the pulsed mode requires a very precise positioning of the fiber after each pullback and the duration of the treatment is much longer. For these reasons, continuous irradiation seems to be preferred by most clinicians. This model should serve as a useful tool to simulate and better understand the mechanism of action of the ELT

## Introduction

Lower-extremity venous insufficiency is a common medical condition afflicting 25% of women and 15% of men in the United States and in Europe. Great saphenous vein (GSV) reflux is the most common underlying cause of significant varicose veins. Traditional treatment of GSV reflux has been surgical removal of the GSV. Although surgical ligation and stripping of the GSV has been the most durable treatment, it is associated with significant peri-operative morbidity. Less-invasive surgical treatments including high ligation of the GSV at the saphenofemoral junction (SFJ) have been attempted in the hope that gravitational reflux would be controlled while the vein is preserved for possible use as a bypass graft. Unfortunately, ligation of the GSV alone usually results in recurrent varicose veins. Even when high ligation has been combined with phlebectomy of varicose tributaries or retrograde sclerotherapy, recurrence has been the rule. Therefore, when it is determined that GSV reflux is the principal underlying problem, treatment should involve eliminating this source of reflux with ablation of any associated incompetent venous segments [1].

In an attempt to reduce morbidity and improve recovery time, several minimally invasive techniques have been developed as alternatives to surgery in the last few years. Endovenous laser treatment (ELT) is one of the most promising of these new techniques [2-4]. In 1999, Boné first reported the delivery of endoluminal laser energy [5].

Numerous studies have since demonstrated that this technique is both safe and efficacious. Several wavelengths have been proposed, respectively 810, 940, 980, 1064 and 1320 nm [6-10] with 810, 940 and 980 the most commonly used. At these wavelengths, power is usually set between 10 and 15 W. The energy is administered endovenously, either in a pulsed fashion (pulse duration: 1 to 3 s with fiber pull back in 3 to 5 mm increments every 2 seconds) or continuously with a constant pullback of the laser fiber (pullback velocity ranging from 1 to 3 mm). At these parameters, doses applied range from 20 J/cm to 140 J/cm [11,12]. These doses induce an heating of the vein wall which is necessary to cause collagen contraction and destruction of endothelium. This stimulates vein wall thickening leading to luminal contraction, venous thrombosis and vein fibrosis [13]. Since tumescent anesthesia is always delivered, patients feel no pain during endovenous laser ablation at the suggested or commonly used laser parameters. The pain that patients feel occurs 5–8 days following the procedure and is related to the inflammation resulting from a successful endovenous ablation (i.e. wall thickening). It is not related to the presence or degree of ecchymosis nor is it the result of non-target laser damage to perivenous tissue. However, if greater doses of energy are delivered, the treatment is becoming painful. Excessive and nonspecific thermal damage has led to 67% of patients complaining of pain along the treated vein for 1 week in one study using the 940-nm intravascular laser [14].

Consequently, successful ELT must achieve permanent damage of the vessel wall, with both the endothelium (tunica intima) and also the outer layer (tunica externa) requiring sufficient heating. Conversely, to avoid side effects "collateral" damage of the perivenous tissue must be avoided. In this context, reports concerning temperatures reached inside vessel lumen during ELT are controversial. In the model studied by Zimmet and Min, peak temperatures (maximum: 50°C) of perivenous tissues generated during ELT (810 nm, 12 W, 1.5s-pulse duration) were unlikely to cause permanent damage to the perivenous tissues. Using another experimental model, Weiss showed that 100% of the laser (810 nm, 12 W, 1s-pulse duration) treated veins showed perforations.

Mathematical modeling of ELT may provide a better understanding of the ELT process and help determine optimal dosage as a function of vein diameter. Although many other models address the laser treatments of blood vessels (Port Wine Stains for example) or Interstitial laser therapy, the mathematical modeling of ELT is yet to be proposed [15-19].

The aim of this paper is to present a mathematical model using dynamic tissue changes based upon the Arrhenius damage model. Numerical simulations are compared to data previously reported in the literature. Optimal parameters emerging from these calculations can be taken in account for improving ELT clinical application.

## Materials and methods

### Mathematical modeling

An opto-thermal model of ELT consists of calculations of light distribution, temperature rise and the extent of thermal damage. The following sections describe the manner in which each stage has been implemented in our calculations.

### Geometrical description of the model

The geometry used to simulate ELT was based on a 2D model consisting of a cylindrical blood vessel (radius: R) including a vessel wall (Thickness: T) and surrounded by infinite and homogenous tissue. Calculations were performed for different vein diameters (3, 5 mm) and at different distances from the center of the vein: in the tunica intima, in the tunica externa and at 1, 2 and 3 mm from the tunica externa.

**Figure 1:**
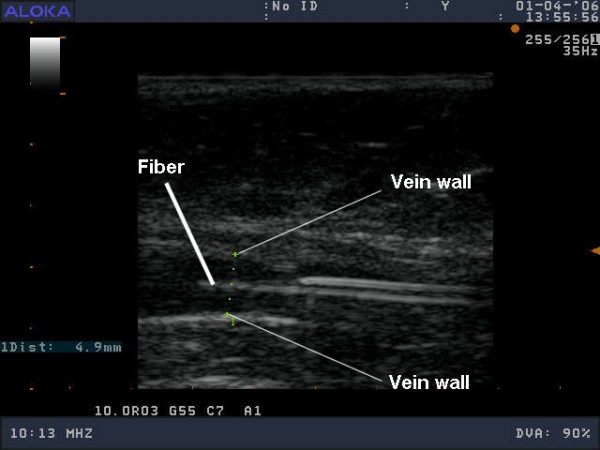
Ultra sound image recorded after tumescent anesthesia. The position of the fiber at the center of the vein is clearly seen (courtesy Dr. Desmyttere and Dr Grard. [27]).

The different vein diameters were chosen since Ultra Sound images recorded after tumescent anesthesia have clearly showed that vein diameters can reach up to 5 mm (figure 1).

**Figure 2:**
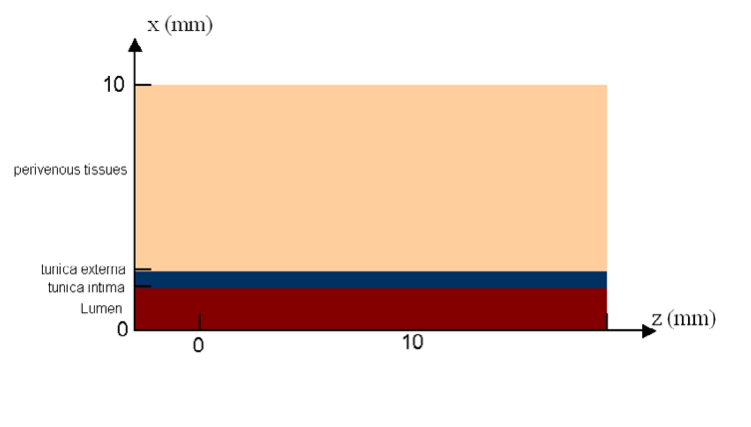
Graphical representation of the geometry used for simulation.

Since, a cylindrical blood vessel is going to be symmetrical from its axis, a 2D section along this axis was considered sufficient for the purposes of calculation (figure 2)

### Light distribution in tissue

The light emitted from the fiber inserted in the vein was modeled as an isotropically radiating point source. As previously proposed by Lizuka et al, spatial distribution has been considered to be dominated by scattering processes [20]. The light irradiance rate (W.mm^-2^) of an isotropic point source emitting P_laser _(W) within an infinite homogeneous medium can be expressed as



where: *P*_*Laser *_(W) : power of the light source

*μ*_*eff *_(mm^-1^) : effective attenuation coefficient

*r *(mm) : radial distance from the source

*D *(mm): optical diffusion distance

*μ*_*eff *_is determined by the following equation



where *μ*_*a *_(mm^-1^) : absorption coefficient in tissue

*μ*'_*s *_(mm^-1^) : reduced scattering coefficient:  = *μ*_*s *_(1 - *g*)

*μ*_*s *_(mm^-1^) : scattering coefficient

*g *: anisotropy factor incorporating the effects of directionally dependent scattering.

*D (mm) *is determined by the following equation:



*r *is defined by the following equation



Where: x (mm): transverse dimension

z (mm): longitudinal dimension

The absorbed power density (W.mm^-3^) is expressed as follows (Welch 1984):

*P*_*abs *_= *μ*_*a*_φ(r)     (4)

The first laser pulse is always applied at coordinates (0,0) in figure 1. When using several pulses, the relative position of the fiber inside the vein is given by:

*z*' = *z *- *z*_*inc*_

Where: z_inc _(mm) is the absolute position of the fiber inside the vein. This position is calculated for each pulse by taking into account the pull-back distance.

When simulating a continuous irradiation performed with a progressive pull-back of the fiber, the relative position of each irradiation is obtained by taking into account the pull-back speed:

*z*_*inc *_= *t *× *v*

where : v (mm.s^-1^) is the pull back speed.

### Calculation of temperature rise

Absorption of light in tissue causes a local elevation in temperature. Tissue heat transfer due to the deposited light is described by the bioheat transfer equation as proposed by Zhang et al [21].



Where

*T *(*r*,*t*): temperature (°K)

ρ : density of tissue (g mm^-3^)

*C *: specific heat of tissue (J. g^-1^.°K^-1^)

*C*_*p *_= *C*·ρ: *heat capacity *(J.mm^-3^.°K^-1^)

*k *= thermal conductivity of tissue (W. mm^-1^.°K^-1^)

*r *= radial distance (mm)

*t *= time (s)

Values used for calculation are reported in Table 1

**Table 1:** listing of physical parameters used for numerical simulation

	Blood	Vessel wall	Perivenous tissue	References
*μ*_*a *_(mm^-1^) Deoxy-hemoglobin	0.20 (810 nm) 0.28 (980 nm)	0.1	0.030	[32,33]
*μ*'_*s *_(mm^-1^)	0.70 (810 nm) 0.6 (980 nm)	2.0	1.0	[32,33]
*μ*_*eff *_(mm^-1^)	0,86 (980 nm)	0.79	0.30	[32,34]
*C *(J.g^-1^.K^-1^)	3.82	3.78	3.78	[33,34]
*ρ *(g.mm^-3^)	1.05.10^-3^	1.05.10^-3^	1.05.10^-3^	[33,34]
*k *(W.mm^-1^.K^-1^)	5.6.10^-4^	5.6.10^-4^	5.6.10^-4^	[33,34]
E_a _(J.mol^-1^)	4.48.10^5^	4.30.10^5^	4.30.10^5^	[34,35]
A (s^-1^)	7.6.10^66^	5.6.10^63^	5.6.10^63^	[34,35]

### Phase transition of blood

In our analysis, we considered that the maximum temperature of blood would not exceed 100°C for all laser parameters. Water being the main constituent of blood, the latent heat of water was included in the calculations. It takes 4.18 J to raise the temperature of one gram of water by one degree C. When considering a 50 mm^3 ^cylinder (diameter: 4 mm, length: 4 mm), the energy required to reach 100°C is 13 J. The latent heat of vaporization of water is 2260 kJ/kg at 100°C. The relationship between pressure and temperature is given by the phase transition diagram of water. At normal pressure: 1.10^5 ^Pa and body temperature 37°C, water is in a liquid state. When the temperature increases, two phenomena can be observed: 1) pressure increases and the water stays in its liquid state, 2) pressure remains constant and water reaches its gaseous state, (steam or vapor) [22] To reach the gaseous state of this 50 mm^3 ^cylinder, (steam or vapor) 119 J will be required. It is therefore unlikely that the blood will boil during ELT.

If we consider the vessel to be a closed but deformable container, water evaporation will not occur, and consequently, water will return to its initial liquid state as soon as the temperature drops back to below 100°C. In conclusion, the assumption is that blood temperature stays around 100°C during laser irradiation is both valid and confirmed by previous studies [23].

### Damage function

Thermal damage in cells and tissue can be described mathematically by a first-order thermal-chemical rate equation, in which temperature history determines damage. Damage is considered to be a unimolecular process, where native molecules transform into a denatured/coagulated state through an activated state leading to cell death. Damage is quantified using a single parameter Ω, which ranges on the entire positive real axis and is calculated from the Arrhenius law [24]. Damage Ω is dimensionless, exponentially dependent on temperature, and linearly dependant on time of exposure.



where *A *(s^-1^) is the frequency factor,

*E*_*a *_(J. mole^-1^) is the activation energy,

*R *(J. mole^-1^.°K^-1^) is the universal gas constant,

The activation energy *E*_*a *_and the frequency factor *A *are derived from thermodynamic variables. They describe the denaturation process of proteins and other cellular constituents. *A *ranges from 10^40^s^-1 ^to 10^105^s^-1^, and *E*_*a *_from 10^5^J/mole to 10^6^J/mole [25]. Values used for calculations are reported in Table 1.

*T *(°K) is the temperature.

Ω can be determined by the following equation



where: *C*_0 _is the concentration of the undamaged molecules at the beginning

*C(t) *is the concentration of the undamaged molecules at time *τ*.

The equation indicates that the measure of damage describes the probability of tissue being destroyed. It is the logarithm of the ratio of the initial concentration of undamaged tissue to the concentration once damage has accumulated, for the time interval t = 0 to t = *τ*. Therefore, Ω = 1 corresponds to an irreversible damage of 100% of the affected cells.

### Numerical implementation

The mathematical model was implemented using the Macsyma-Pdease2D software (Macsyma Inc., Arlington, MA, USA). This Finite Element CAD Software needs to specify the Partial Differential Equations, variables, geometry, and boundary conditions; PDEase2D creates both numerical output tables and plots. PDEase2D generates and refines the element grid, adaptively selects time steps, and iterates until it attains convergence in nonlinear problems. And because PDEase2D does automatic error analysis, you do not need to make several runs with different meshes to verify convergence. You can choose whether or not to override the automatic defaults

For numerical simulations, parameters commonly used for ELT were used. A 600 μm laser fiber was considered. Two different vessel diameters were evaluated: 3 mm and 5 mm. Laser power (10 & 15 W), pulse duration (1, 2, 3 s), delay (or off-phase) between pulses (2, 3 and 4 s) and pullback distances (3, 5 and 7 mm) were the variables. In this case, thermal energy is applied along the length of the vein by withdrawing the laser fiber in 3, 5 or 7 mm increments.

For CW mode simulation, laser power (10, 15 W) and pullback speed (1,2 and 3 mm/s) were the variables.

An irregular 10 × 60 finite element grid was used. The time steps were 0.1s. The tolerances used to converge the solution were 10^-3^. The vein wall thickness was considered to be 0.4 mm. The initial temperature was set at 37°C. The listing of physical parameters used for numerical simulation is reported in Table 1.

The product of pullback rate and power yields the total dose of energy delivered to a vein during treatment. Several studies have suggested that this parameter is a major determinant of treatment outcome [26-28]. In order to compare the results obtained through mathematical modeling, the energy per centimeter (J/cm) was calculated.

## Results

### Light distribution

**Figure 3:**
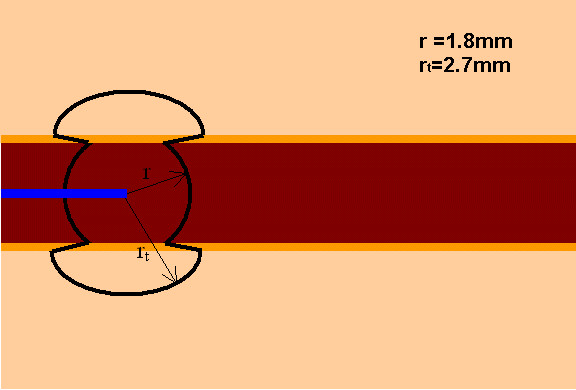
Light distribution inside and outside a 3 mm vein when using a 600 μm fiber. The line illustrates where 10% of the initial light irradiance rate is obtained.

Figure 3 illustrates how the light is distributed inside and outside a 3 mm vein when using a 600 μm fiber. The line illustrates where 10% of the initial light irradiance rate is obtained.

### Wavelength

**Figure 4:**
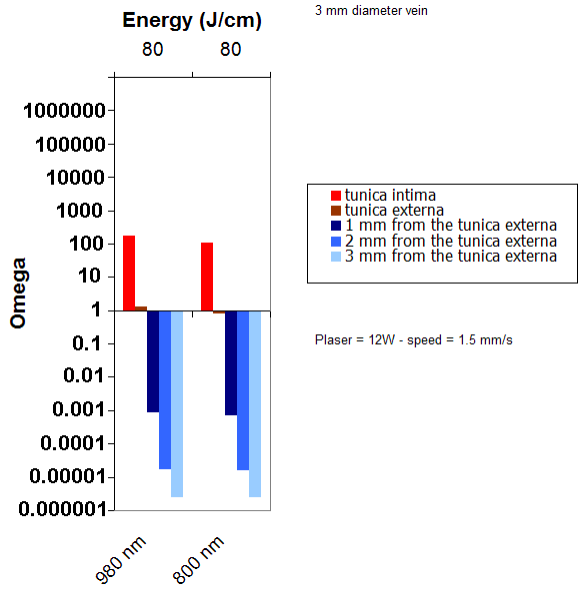
Damage obtained for two different wavelengths (800 nm and 980 nm) power: 12 W, CW, pullback speed : 1.5 mm/s, Energy: 80 J/cm, vein diameter: 3 mm.

The role of the laser wavelength was investigated. Figure 4 presents the results of mathematical modeling for 810 and 980 nm, using the same energy of 80 J/cm. Due to its greater absorption in blood, 980 nm leads to slightly greater damage of the tunica intima.

### Pulsed mode

**Figure 5:**
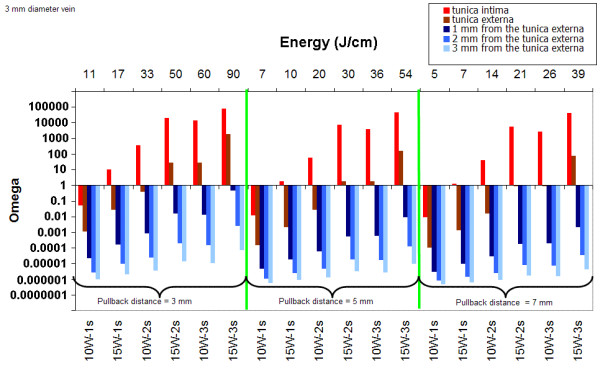
Damage as a function of power, pulse duration and pull back distance for a 3 mm vein diameter (delay between pulses: 2s, λ = 980 nm).

**Figure 6:**
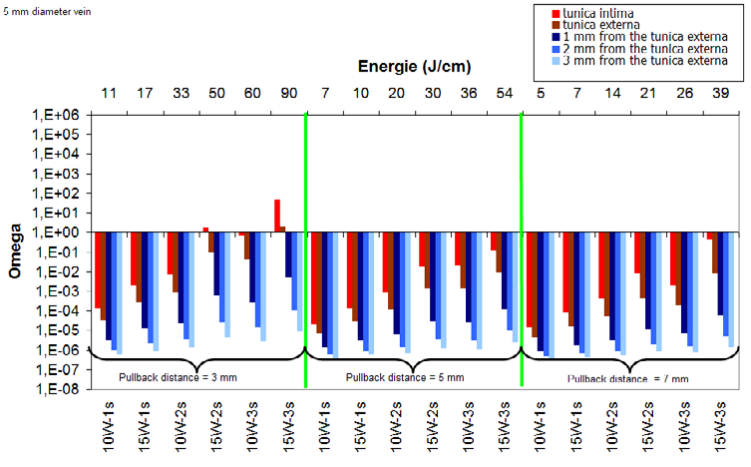
Damage as a function of power, pulse duration and pull back distance for a 5 mm vein diameter (delay between pulses: 2s, λ = 980 nm).

Figure 5 (3 mm vein diameter) and Figure 6 (5 mm vein diameter) summarize the results of mathematical modeling for the different set of parameters. For a 3 mm vein diameter, irrespective of the pullback distance (2, 5 or 7 mm), at least 15 J/cm is required to achieve permanent damage of the tunica intima. For a 5 mm vein diameter, permanent damage of the tunica intima is achieved at 15 W and when using a 3 mm pullback distance. A power level of 15 W delivered for 2 s (50 J/cm) or 3s (90 J/cm) is efficacious. A power level of 10 W is too low to lead to permanent damage of the intima.

**Figure 7:**
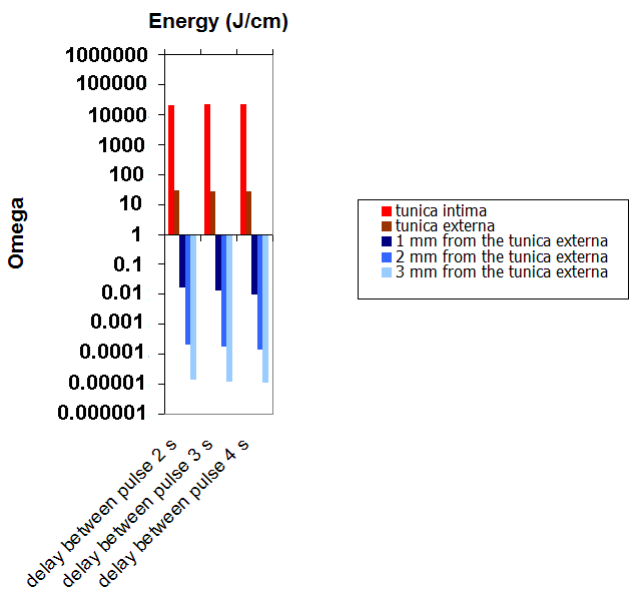
Damage as a function delay between pulse (2s, 3s and 4s). Power: 15 W, pulse duration: 2s ; vein diameter: 3 mm; λ = 980 nm).

Figure 7 shows that the delay between pulses has a very limited effect on wall damage since heat convection play a minor role on heat transport. For a 3 mm diameter vein, energy between 30 J/cm up to 50 J/cm seems to be optimal to provide selective damage to the vessel wall.

**Figure 8:**
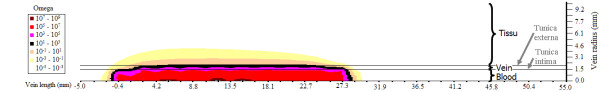
Isodamage distribution inside tissues: power: 15 W, pulse: 2s, delay: 2s, pull-back distance: 3 mm ; vein diameter: 3 mm; λ = 980 nm).

**Figure 9:**
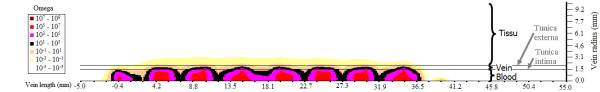
Isodamage distribution inside tissues power: 15 W, pulse: 2s, delay: 2s, pull-back distance: 5 mm; vein diameter: 3 mm, λ = 980 nm).

**Figure 10:**
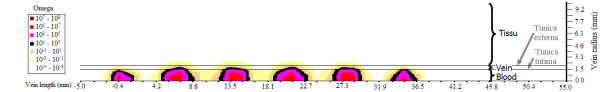
Isodamage distribution inside tissues power: 15 W, pulse: 2s, delay: 2s, pull-back distance: 5 mm; vein diameter: 3 mm, λ = 980 nm).

Figures 8, 9 and 10 display some examples of damage distribution at different sets of parameters. These figures clearly show that pull-back distance plays a major role when determining the energy applied per centimeter.

When using 3 mm increments, the vein is damaged homogenously along its length. When using 5 mm increments, damage is less homogeneous even though the tunica intima is always damaged. Finally, for 7 mm increments, it is evident that the vein is damaged sequentially along the entire length and consequently the energy applied is lower. Cinepak movies of real time isodamage distribution are also provided for figures 8, 9 and 10 (see additional files 1, 2 and 3).

### CW mode

**Figure 11:**
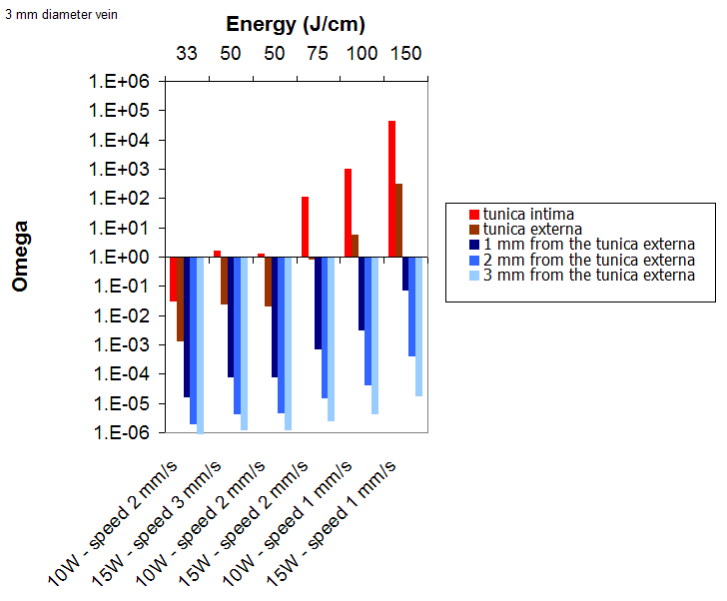
Damage as a function as a function of power and pullback speed for a 3 mm diameter vein; λ = 980 nm.

Figure 11 displays the results obtained for several set of parameters for a 3 mm vein diameter. Simulations show that for 10 W and 2 mm/s pull-back speed, the tunica intima is damaged (50 J/cm). A minimum of 100 J/cm is required to obtain a permanent damage of the vessel wall.

**Figure 12:**
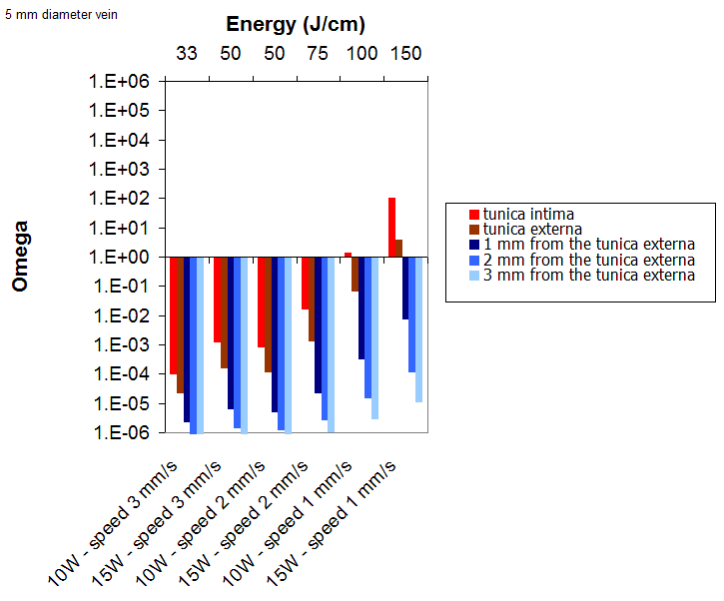
Damage as a function as a function of power and pullback speed for a 5 mm diameter; λ = 980 nm.

Figure 12 displays the results obtained for several set of parameters for a 5 mm vein diameter. Simulations show that for 15 W and 2 mm/s pull-back speed, the tunica intima remains undamaged. A minimum of 100 J/cm is required to obtain a permanent damage of the intima and 150 J/cm to damage the vessel wall.

**Figure 13:**
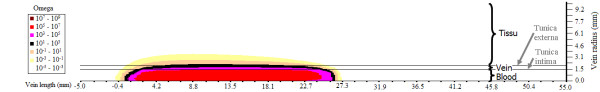
Isodamage distribution inside tissues : power: 15 W, pull-back speed: 1.5 mm/s, vein diameter: 3 mm, λ = 980 nm).

Figure 13 display one example of damage distribution for CW (Power: 15 W, pullback speed: 1.5 mm/s, Vein diameter: 3 mm). This figure shows that the vein is damaged homogenously along its length Cinepak movie of real time isodamage distribution is also provided for figure 13 (see additional file 4)

## Discussion

To date, mathematical modeling of ELT has never been proposed. This task was performed to assist in providing a better understanding of the ELT process and possibly to determine the optimal dosage as a function of vein diameter. Our model remains a mathematical model, implying that errors may appear owing to the considerations and simplifications required to realize it. Generally, such errors appear because of inaccuracy of the optical, thermal, and damage properties that are critical points in the model's set of equations. In fact, these properties play a key role in the accuracy of the results achieved. Many methods have been presented to calculate these properties but still we see differences in the values presented by the different groups, which reflect the difficulty of measuring these properties. The problem is increased by the reliance of the properties on different variables (temperature, damage) over time. This makes the deviation neither linear nor regular [24].

Before attempting to compare the parameters used for simulation to those usually reported in the literature, the following comments must be made:

1) One of the main problems remains the knowledge of the vein diameters during ELT treatment. Since the vein diameter is considerably reduced after tumescent anesthesia, it should be systematically measured. A recent study by Desmyttere et al have demonstrated that after tumescent anesthesia, the vein diameter was usually reduced down to 5 mm or less [27].

2) In contrast to the mode of action of VNUS closure (radiofrequency) where a significant shrinkage of the vessel wall is observed, Proebstle has clearly demonstrated that, when performing ELT, permanent occlusion, reported at 3 months or later, can be obtained by thermal damage of the tunica intima inner vein wall only [29]. This observation is confirmed by the histological study performed by Corcos et al. They showed that when permanent occlusion was observed, the endothelium and intima were always damaged and that success was independent of the vessel wall thickness [30].

The results of mathematical modeling for 810 and 980 nm shows 980 nm leads to a slightly greater damage of the tunica intima when compared to 810 nm. This is owed to its better absorption by blood at 980 nm. However, because of the inaccuracy on vein diameter, one can consider that the choice of the wavelength between 810 and 980 nm has no influence on the results. This is confirmed by the literature where 810, 940, 980 nm were used for ELT with similar parameters [7,9,10]. Proebstle et al performed an in vitro study to evaluate the role of intravascular blood for the effective transfer of thermal damage to the vein wall through absorption of laser energy with 810, 940 and 980 nm. Similar results were obtained with 810 nm, 940 nm and 980 nm [29].

When using the continuous mode (810 nm, 14 W), Timperman, has treated 100 veins with an average energy of 95 J/cm (range, 57–145 J/cm; SD: 16 J/cm). Follow-up and success at 1 week was 100%, 96% at 3 month follow-up and finally 95% at 9 month follow-up [31]. Using 58 J/cm, in another series, Timperman reported only a 76% complete vein ablation rate. Similarly, Theivacumar has confirmed that, of all parameters, energy per cm was the main determinant of successful LSV ablation by ELT [26]. The parameters used by Timperman are very similar to those determined by our simulations. In continuous mode, for a 3 mm vein diameter, 50 J/cm are required to damage the vessel wall. For a 5 mm diameter, 100 J/cm are required.

In pulsed mode, it is often difficult to obtain details concerning parameters used for ELT. If power and pulse duration are usually reported, information concerning speed of fiber withdrawal is usually missing. Results reported in the literature are highly variable and tend to prove that this parameter is not well controlled by the operator. In a study performed in 476 limbs (810 nm, 12 W, 1s and a pull back distance varying from 2 mm to 2.8 mm giving respectively 61 J/cm and 43 J/cm), Theivacumar et al observed that occlusion rates were significantly greater at higher energy levels [26]. Using 50 J/cm, in a series of 56 limbs in 41 patients, Mozes et al reported complete resolution at 3-month follow up [11]. Finally, Proebstle et al on a series of 77 patients, performed ELT with a median energy delivery of 23.4 J/cm (range of 11.8 to 35.5) Using these parameters, at 3 months post ELT, 10% of GSVs were found open by color Doppler examination [12]. These results are similar to our calculations in that it was determined that 15 J/cm is required to obtain a permanent damage of a 3 mm vein diameter and 50 J/cm for a 5 mm vein diameter.

An interesting observation is that less energy is required in pulsed mode than in continuous mode because the vein is not heated along its entire length. However, as illustrated in figures 9 and 10, damaging the vein sequentially along its entire length may lead to permanent occlusion.

At last, if steam formation during ELT has already been reported, the interpretation given by the authors is inappropriate [6]. Steam bubbles originating from boiling blood cannot be the pathophysiological mechanism of action of ELT. The steam produced by absorption of laser energy by the blood is a tiny fraction of the energy necessary to damage the vein wall and cannot be the primary mechanism of injury to the vein with endovenous laser. The carbonization and tract within the vein walls seen by histology following endovenous laser can only be the result of direct contact between the laser fiber tip and the vein wall. Venous caliber reduction is maybe due to collagen shrinking by simple heating, but media contraction is only obtained by selective 810–980 nm irradiation.

Consequently, the parameters determined through calculation in our model, especially the ones concerning the different outcome in relation with the different diameters, seem to confirm and explain the observations emerged by the clinical practice

## Conclusion

The parameters determined by mathematical modeling are in agreement with those used in clinical practice. They confirm that thermal damage of the inner vein wall (tunica intima) is required to achieve the tissue alterations necessary in order to lead the vein to permanent occlusion. However, in order to obtain a high rate of success without adverse events, the knowledge of the vein diameter after tumescent anesthesia is recommended in order to use the optimal energy. As clearly demonstrated by our calculations, both pulsed and continuous mode operations of the laser can be efficient. An interesting observation in our model is that less amount of energy is required in pulsed mode than in continuous mode. Damaging the vein sequentially along its entire length may lead to permanent occlusion. However, the pulsed mode requires a very precise positioning of the fiber after each pullback and the duration of the treatment is much longer. For these reasons, continuous irradiation seems to be preferred by most clinicians.

This model should serve as a useful tool to simulate and better understand the mechanism of action of the ELT

## Supplementary Material

Additional File 1 The movie shows the isodamage distribution inside tissues using the following parameters: power: 15 W, pulse: 2s, delay: 2s, pull-back distance: 3 mm ; vein diameter: 3 mm; λ = 980 nm. The movie belongs to Figure 8. The file can be played using the internet browser. Click here for file

Additional File 2 The movie shows the isodamage distribution inside tissues using the following parameters: power: 15 W, pulse: 2s, delay: 2s, pull-back distance: 5 mm ; vein diameter: 3 mm; λ = 980 nm. The movie belongs to Figure 9. The file can be played using the internet browser. Click here for file

Additional File 3 The movie shows the isodamage distribution inside tissues using the following parameters: power: 15 W, pulse: 2s, delay: 2s, pull-back distance: 7 mm ; vein diameter: 3 mm; λ = 980 nm. The movie belongs to Figure 9. The file can be played using the internet browser. Click here for file

Additional File 4 The movie shows the isodamage distribution inside tissues using the following parameters: : power: 15 W, pull-back speed: 1.5 mm/s, vein diameter: 3 mm, λ = 980 nm. The movie belongs to Figure 13. The file can be played using the internet browser. Click here for file
